# Knowledge, beliefs, perceived barriers, and previous upper gastrointestinal endoscopy use regarding *Helicobacter pylori* and gastric cancer prevention among community residents: a cross-sectional study

**DOI:** 10.3389/fpubh.2026.1912334

**Published:** 2026-08-14

**Authors:** Huiying An, Xue Wang, Yan Chen, Lei Lei

**Affiliations:** 1 Department of Gastroenterology, Henan Provincial People’s Hospital, Zhengzhou, Henan, China; 2 Department of Gastroenterology, People’s Hospital of Zhengzhou University, Zhengzhou, Henan, China

**Keywords:** cross-sectional study, gastric cancer prevention, *Helicobacter pylori*, knowledge-attitude-practice, perceived barriers, upper gastrointestinal endoscopy

## Abstract

**Background:**

Low adherence to primary and secondary prevention strategies for gastric cancer remains a major public health challenge. Understanding knowledge, beliefs, perceived barriers, and previous upper gastrointestinal endoscopy (UGE) use may help identify modifiable targets for prevention programs.

**Objective:**

This study aimed to describe knowledge, beliefs, perceived barriers, and previous UGE use related to *Helicobacter pylori* infection and gastric cancer prevention among community residents in Henan Province, China, and to examine their associations.

**Methods:**

A descriptive cross-sectional knowledge-attitude-practice survey was conducted among 1,911 community residents in Henan Province, China. Multivariable logistic regression was used to estimate adjusted odds ratios for previous UGE use. Bayesian path analysis was performed to explore indirect associations among knowledge, beliefs, perceived barriers, and previous UGE use. All results were interpreted as associations rather than causal effects.

**Results:**

Overall, 25.1% (480/1,911) of participants reported previous UGE use, and 15.3% reported a history of *H. pylori* infection. Previous UGE use was more common among participants with self-reported *H. pylori* infection (adjusted odds ratio [aOR] 2.29, 95% CI 1.70–3.08), a first-degree family history of gastric cancer (aOR 4.65, 95% CI 3.14–6.89), and gastrointestinal symptoms (aOR 1.67, 95% CI 1.32–2.10). Higher belief scores were associated with lower odds of previous UGE use (aOR 0.29, 95% CI 0.16–0.51). In Bayesian path analysis, knowledge was positively associated with beliefs (*β* = 0.502, *p* < 0.001) and previous UGE use (*β* = 0.126, p < 0.001), whereas perceived barriers were negatively associated with previous UGE use (*β* = −0.220).

**Conclusion:**

Previous UGE use remained suboptimal despite generally favorable awareness and beliefs. These findings suggest an important gap between cognitive readiness and preventive action. Public health strategies should combine targeted health education with barrier reduction, risk-based referral, physician recommendation, and integration of *H. pylori* screen and treat strategies.

## 1 Introduction

Gastric cancer (GC) remains a major public health burden worldwide. According to GLOBOCAN 2024, stomach cancer was estimated to cause 980,286 new cases and 641,554 deaths worldwide in 2024, ranking fifth for both incidence and mortality (1). China continues to bear a substantial proportion of the global burden, with national cancer statistics showing that GC remains among the leading causes of cancer incidence and death (2). The public health importance of prevention is reinforced by the strong stage-survival gradient: the 5-year relative survival rate is markedly higher for localized disease than for distant-stage disease (3). These data highlight the need for prevention strategies that promote early risk identification, timely treatment of modifiable risk factors, and endoscopic evaluation among appropriate high-risk groups.

*Helicobacter pylori* (*H. pylori*) infection is a major modifiable risk factor for non-cardia GC. Eradication therapy reduces subsequent GC risk, particularly when implemented before advanced precancerous changes have developed (4, 5). Current prevention thinking has therefore shifted from passive treatment of symptomatic infection toward proactive *H. pylori* screen-and-treat strategies. The 2025 International Agency for Research on Cancer (IARC) guidance and the 2026 IARC Working Group Report in the New England Journal of Medicine emphasize population-based *H. pylori* screen-and-treat programs as an evidence-informed approach for GC prevention, while also stressing quality assurance, antibiotic stewardship, feasibility, acceptability, and equity in implementation (6, 7).

Upper gastrointestinal endoscopy (UGE) is central to secondary prevention because it enables direct mucosal visualization, biopsy, diagnosis of precancerous lesions, and surveillance of high-risk individuals. Evidence from Asian studies and community-based trials supports the association between endoscopic screening and reduced GC or upper gastrointestinal cancer mortality (8–10). International guidance increasingly recommends risk-based endoscopic screening and surveillance, including assessment and follow-up of gastric atrophy and intestinal metaplasia using endoscopic and histologic staging systems (11, 12). In China, current national recommendations use a risk-based rather than universal screening approach. Adults aged 45 years or older who have at least one additional risk factor—such as residence in a high-incidence area, *H. pylori* infection, a first-degree family history of GC, adverse dietary or lifestyle factors, or specified gastric diseases—are classified as a high-risk population. Endoscopic screening is generally recommended for eligible high-risk individuals aged 45–74 years (13, 14). Thus, 45 years represents the lower age boundary for guideline-based risk assessment, but age alone does not establish screening eligibility.

Despite these advances, adherence to GC prevention strategies remains fragmented. Many individuals do not complete *H. pylori* testing, eradication confirmation, risk stratification, or UGE screening even when they have risk factors or favorable attitudes. Previous studies have identified multiple barriers, including symptom-oriented perceptions, fear of endoscopic discomfort, financial burden, lack of physician recommendation, uncertainty about where to seek screening, and sociodemographic disparities in access (15–18). These barriers are particularly important in community settings, where many individuals are asymptomatic and may not perceive GC prevention as an urgent health priority.

This study was guided by an expanded Knowledge-Attitude-Practice (KAP) framework. The KAP model has been used in gastric cancer prevention research to examine how knowledge shapes attitudes or beliefs and how these factors may influence health-related behaviours (19). However, cancer prevention and screening behaviours are rarely determined by knowledge alone. Individuals may be aware of gastric cancer risk factors, *H. pylori*-related prevention, or screening recommendations, but still fail to engage in preventive behaviours because of psychological, practical, or health-system barriers. To address this limitation, the present study incorporated key constructs from the Health Belief Model, including perceived susceptibility, perceived benefits, perceived barriers, cues to action, and self-efficacy (20). In gastric cancer prevention, barriers such as absence of symptoms, fear of endoscopic discomfort, cost, lack of time, and insufficient physician recommendation may interrupt the transition from knowledge and beliefs to behavioral intention or actual screening uptake (16, 21). This descriptive cross-sectional study therefore aimed to describe knowledge, beliefs, perceived barriers, and previous UGE use related to *H. pylori* infection and GC prevention among community residents in Henan Province, China, and to examine their associations using multivariable regression and Bayesian path analysis.

## 2 Materials and methods

### 2.1 Study design and participants

This study used a descriptive cross-sectional KAP design to assess knowledge, beliefs, perceived barriers, and previous UGE use related to *H. pylori* infection and GC prevention among community residents in Henan Province, China. Because of the cross-sectional design, all analyses were intended to describe prevalence and examine associations rather than to infer causality or temporal relationships. Data were collected between January and December 2025 using an online self-administered questionnaire distributed via Wenjuanxing, which required completion of all mandatory items before submission.

Participants were recruited using a combination of convenience and snowball sampling. The survey link was disseminated through community networks, social media, and local health-promotion channels. Participants were encouraged to share the link with family members, friends, or colleagues. Eligible participants were residents aged 18 years or older who provided informed consent. The specific objectives were: (1) to assess knowledge and beliefs regarding *H. pylori* infection and GC prevention; (2) to describe perceived barriers to UGE use; and (3) to examine associations among knowledge, beliefs, perceived barriers, and previous UGE use.

### 2.2 Calculation of sample size

The required sample size was estimated according to the main binary outcome: previous UGE use versus no previous UGE use. Assuming an expected screening prevalence of approximately 25%, a two-sided alpha level of 0.05, 80% power, and an odds ratio of 1.5 for key explanatory variables, the minimum required sample size was increased to account for multivariable modeling and planned path analysis. The sample size was also considered adequate for structural equation/path modeling according to commonly cited recommendations that sample-size requirements depend on model complexity, factor loading strength, and the number of estimated parameters (22). Ultimately, 1,911 completed questionnaires were included in the final analysis. The odds ratios reported in this study should be interpreted as measures of association with the odds of previous UGE use, not as risk ratios or causal effects.

### 2.3 Statistical analysis

All statistical analyses were performed using SPSS 26.0 (IBM Corp., Armonk, NY, USA) and Mplus 8.5 (Muthen & Muthen, Los Angeles, CA, USA). Continuous variables are presented as means and standard deviations, and categorical variables as counts and percentages. Associations between participant characteristics and previous UGE use were evaluated using Pearson chi-square tests for categorical variables and independent-samples *t* tests or one-way analysis of variance for continuous variables, as appropriate.

Age was categorized *a priori* as <45 and ≥45 years because current Chinese guidance identifies 45 years as the lower age boundary for risk-based GC screening (13, 14). This dichotomy was intended to distinguish participants who had reached the guideline starting age from younger adults; it was not intended to imply that age alone determines screening eligibility. The prespecified candidate explanatory variables for multivariable logistic regression were age group, sex, marital status, education level, annual household income, residence, self-reported *H. pylori* infection, *H. pylori* infection among family members, gastrointestinal symptoms, a first-degree relative with GC, a friend or colleague with GC, use of serving chopsticks or separate dining at home and when eating out, the knowledge score, and the belief score. These variables were selected according to the expanded KAP/Health Belief Model framework and previous evidence on sociodemographic access, clinical risk and cues to action, family and social exposure, preventive behavior, and cognitive factors (16–21, 23–26). All variables were entered simultaneously using the Enter method; no automated stepwise selection or univariate *p*-value screening was used.

The perceived barrier score was not included in the logistic regression because it was conceptualized as an intermediate construct in the Bayesian path model rather than as a baseline covariate. Binary logistic regression was used for the main binary outcome, and adjusted odds ratios (aORs) with 95% confidence intervals (CIs) were reported. Bayesian path analysis was conducted in Mplus to explore theoretically informed indirect associations among knowledge (X), beliefs (M1), perceived barriers (M2), and previous UGE use (Y), following published Mplus Bayesian estimation guidance (27). A serial indirect pathway (X → M1 → M2 → Y) was examined. Model fit was evaluated using the posterior predictive *p*-value (PPP) and the 95% credibility interval for the chi-square difference. Path coefficients are reported as unstandardized (B) and standardized (*β*) estimates with standard errors, 95% intervals, and *p*-values. Because the data were cross-sectional, odds ratios and indirect pathways were interpreted as associations rather than causal effects.

### 2.4 Questionnaire

#### 2.4.1 Questionnaire development

The questionnaire was developed following the World Health Organization guide to developing KAP surveys (28) and practical guidance for KAP instrument development (29). An initial item pool was generated based on literature review, clinical expertise, and the study objective. It consisted of four sections: sociodemographic characteristics, medical history and exposure, lifestyle behaviors, and KAP regarding *H. pylori* infection and GC prevention. The sociodemographic section collected information on age, sex, ethnicity, marital status, education, occupation, income, residence, and health insurance status. The medical history and exposure section captured recent gastrointestinal symptoms, diagnosed diseases, previous UGE use, self-reported *H. pylori* infection status, *H. pylori* treatment and re-examination history, and family history. The lifestyle section assessed dining practices, including the use of serving chopsticks or separate dining at home or when eating out. A multiple-response item assessed perceived barriers to UGE use. This item listed 12 common barriers, including fear of discomfort, absence of symptoms, cost, lack of knowledge about screening benefits or locations, and lack of physician recommendation. The item was designed to describe the cumulative burden of perceived barriers and was not intended to form a reflective psychometric scale.

#### 2.4.2 Content validity and pilot testing

To ensure content validity, the initial item pool was evaluated by a panel of five experts in gastroenterology, epidemiology, and health education across two rounds of review, following standard expert-validation procedures for KAP instruments. Items were revised according to expert feedback. The scale-level content validity index indicated excellent agreement (S-CVI/UA > 0.80). A cognitive debriefing pilot test was then conducted among 30 community residents to assess item clarity, wording, and comprehensibility before formal field implementation.

#### 2.4.3 Construct validity and reliability

Psychometric properties of the knowledge and belief dimensions were evaluated in a pilot study involving 500 community residents independent of the main survey sample. Exploratory factor analysis (EFA) was performed to assess construct validity. For the knowledge domain, data suitability was confirmed (KMO = 0.934; Bartlett test, chi-square = 29,599.95, *p* < 0.001). Based on the screen plot and theoretical interpretability, a four-factor solution using Promax oblique rotation was retained, explaining 41.7% of the cumulative variance. Items with primary loadings of 0.40 or higher and cross-loading differences of 0.20 or greater were retained. The final knowledge structure comprised: (1) awareness of *H. pylori* infectivity (2 items, loadings 0.46–0.79); (2) transmission routes (12 items, absolute loadings 0.40–0.68); (3) screening and UGE perceptions (10 items, loadings 0.43–0.73); and (4) risk factors (11 items, loadings 0.57–0.79). Interfactor correlations (r = −0.22 to 0.52) supported the use of oblique rotation.

EFA for the belief scale (5 items) demonstrated a unidimensional structure (KMO = 0.79, *p* < 0.001, eigenvalue = 2.62), explaining 52.4% of the variance with factor loadings of 0.54–0.69. Consistent with KAP scale development, the practice domain was treated as a set of behavioral indicators rather than a reflective latent construct; therefore, it was not subjected to factor analysis. Formal external validation in an independent geographic or international population was not conducted and was added as a limitation.

The instrument demonstrated acceptable internal consistency. For the knowledge scale, Cronbach alpha was 0.893 for the overall scale, with subscale alpha values ranging from 0.868 to 0.926. The belief scale showed acceptable reliability (Cronbach alpha = 0.740).

#### 2.4.4 Scoring

Knowledge items were scored using a Multiple True-False (MTF) format. Each response option was treated as an independent True/False item, with a correct answer scored as 1 and an incorrect or uncertain response as 0. The score for each knowledge sub-domain was then calculated as the mean of all item scores within that sub-domain, yielding a range of 0 to 1.

Belief items were measured on a 3-point Likert scale (1 = disagree, 2 = neutral, 3 = agree). All items were positively worded; negatively phrased items were reverse-coded prior to analysis. To align the scoring metric with the knowledge domain, each belief item score was first normalized to a 0–1 range using the min-max transformation: Score_norm_ = (Score_obs_ − Score_min_)/(Score_max_ − Score_min_). The overall belief score was computed as the mean of the normalized item scores, also ranging from 0 to 1.

Previous UGE use was assessed using the questionnaire item that used the lay term “gastroscopy”: “Have you ever undergone a gastroscopy?” Response options included “Yes,” “No, but I would consider it,” and “No, and I do not plan to consider it.” In the manuscript, this variable was standardized as previous UGE use. Responses were dichotomized as previous UGE use (1 = yes) and no previous UGE use (0 = both no responses).

Perceived barriers were assessed for all participants using a multiple-response item listing 12 common factors derived from literature review and expert consultation. Participants indicated which factors they believed could hinder their willingness to undergo UGE. Each selected barrier was coded as 1 and unselected items as 0. Participants who selected “None of the above” were assigned a Perceived Barrier Score of 0. Because this variable represented the cumulative burden of perceived obstacles rather than a reflective latent psychological construct, a simple additive scoring approach was adopted instead of psychometric scale modeling. The score was standardized to a 0–1 range using min-max transformation before path analysis.

### 2.5 Ethical considerations

Informed consent was obtained electronically from all participants before the survey. Participation was voluntary, and responses were anonymized before analysis. This study was approved by the Ethics Committee of People’s Hospital of Zhengzhou University (No. 53, 2021). During revision of the manuscript, generative artificial intelligence assistance was used only to support language editing, organization, and consistency checking. The authors critically reviewed and verified all AI-assisted text, references, and interpretations. AI tools were not used for data collection, statistical analysis, or generation of scientific conclusions.

## 3 Result

### 3.1 Characteristics of study participants

The sociodemographic and clinical characteristics of the 1,911 participants are summarized in Table 1. The mean age was 37.65 ± 12.17 years. 1,427 participants (74.7%) were aged <45 years and 484 (25.3%) were aged ≥45 years. Females accounted for 58.6% of the sample. Most participants were married (77.1%) and had a college or bachelor’s degree (60.0%). Annual household income was <30,000 CNY in 41.0% of participants, and 54.5% resided in rural areas. The self-reported *H. pylori* infection rate was 15.3%. Gastrointestinal symptoms were reported by 44.4%, a first-degree family history of GC by 7.7%, and a friend or colleague with GC by 18.3%. Overall, 30.1% (575/1,911) reported using serving chopsticks or practicing separate dining at home, and 47.6% (909/1,911) did so when eating out.

**Table 1:** Sociodemographic, clinical, and lifestyle characteristics of study participants (N = 1,911).

Variable	Category	*n*	%
Age (years)	<45	1,427	74.7
	≥45	484	25.3
Gender	Male	791	41.4
	Female	1,120	58.6
Marital status	Unmarried	437	22.9
	Married	1,474	77.1
Education level	Primary school or below	28	1.5
	Junior high school or below	291	15.2
	High school/Technical secondary	330	17.3
	College/Bachelor	1,146	60.0
	Master or above	116	6.1
Annual income (CNY)	<30,000	784	41.0
	30,000–60,000	482	25.2
	60,000–100,000	334	17.5
	>100,000	311	16.3
Residence	Urban	870	45.5
	Rural	1,041	54.5
*H. pylori* infection (self-reported)	No	1,618	84.7
	Yes	293	15.3
Family *H. pylori* infection	Yes	480	25.1
	No	1,431	74.9
Gastrointestinal symptoms	Yes	848	44.4
	No	1,063	55.6
First-degree relative with GC	Yes	148	7.7
	No	1,763	92.3
Friend/colleague with GC	Yes	350	18.3
	No	1,561	81.7
Use of serving chopsticks/separate dining at home	Yes	575	30.1
	No	1,336	69.9
Use of serving chopsticks/separate dining when eating out	Yes	909	47.6
	No	1,002	52.4
Previous UGE use	Yes	480	25.1
	No, but I would consider it	1,027	53.7
	No, and I do not plan to consider it	404	21.2

GC, gastric cancer; CNY, Chinese Yuan; UGE, upper gastrointestinal endoscopy.

### 3.2 Knowledge, beliefs, previous UGE use, and perceived barriers

#### 3.2.1 Knowledge

**Figure 1:**
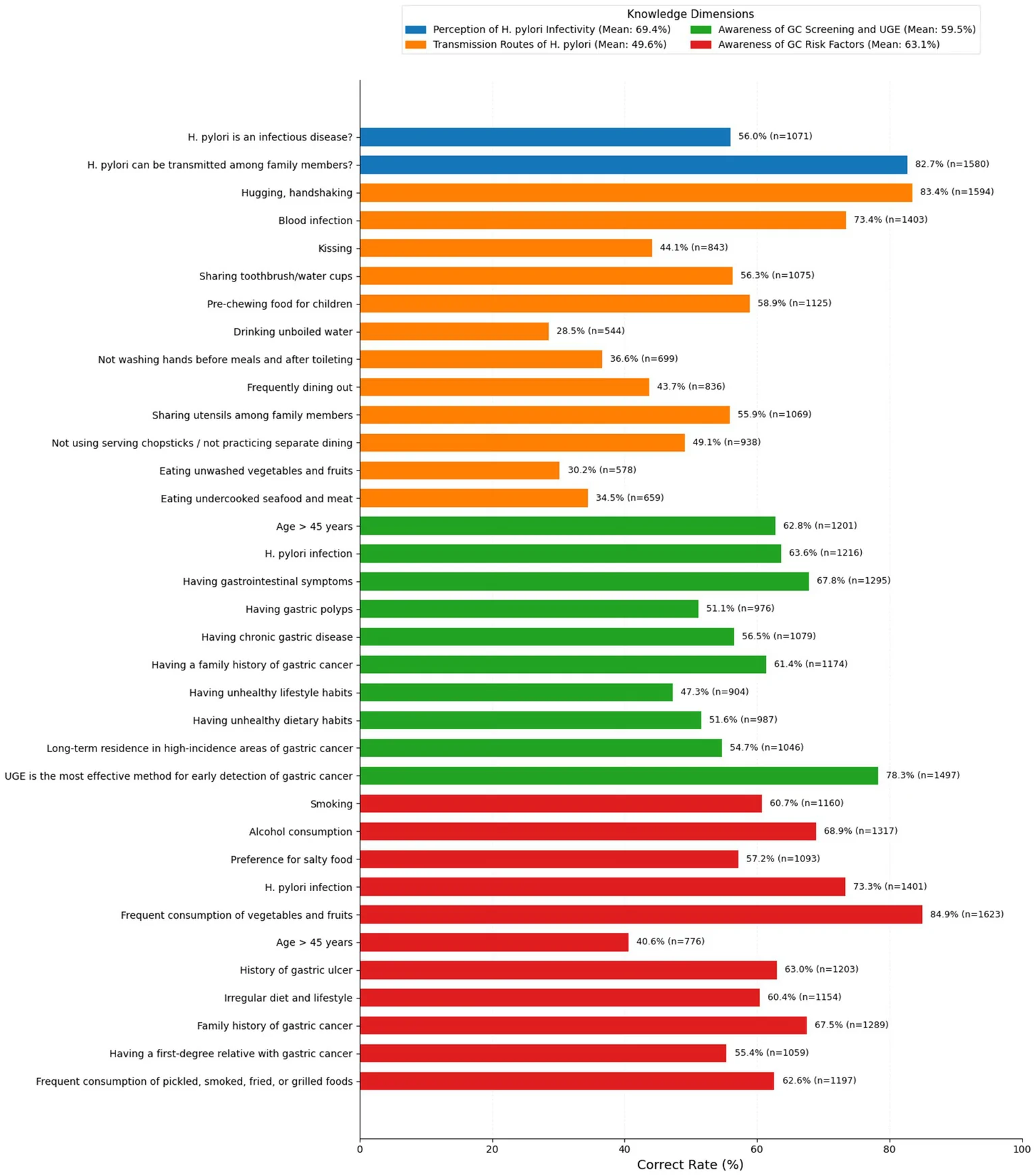
Percentage distribution of correct knowledge rates across multidimensional items regarding *Helicobacter pylori* infection and gastric cancer prevention (*N* = 1,911).

General knowledge regarding *H. pylori* infectivity, *H. pylori* transmission, GC screening, and GC risk factors was suboptimal among participants. The mean correct rates for the four dimensions were 69.4% for awareness of *H. pylori* infectivity, 49.6% for *H. pylori* transmission routes, 59.5% for perceptions of GC screening and UGE, and 63.1% for awareness of GC risk factors. Although most participants knew that *H. pylori* can transmit among family members (82.7%), only 56.0% explicitly considered it an infectious disease. Knowledge of transmission routes showed a gap between correct rejection of casual contact (83.4%) and low recognition of fecal-oral pathways such as drinking unboiled water (28.5%) or eating unwashed produce (30.2%). For screening-related knowledge, UGE was widely endorsed (78.3%), but recognition of specific high-risk groups and risk-based indications varied substantially across items. Concerning risk factors, *H. pylori* infection (73.3%) and protective dietary factors (84.9%) were well recognized, whereas only 40.6% identified age older than 45 years as a risk factor. Figure 1 presents the item-level knowledge results.

#### 3.2.2 Beliefs

**Figure 2:**
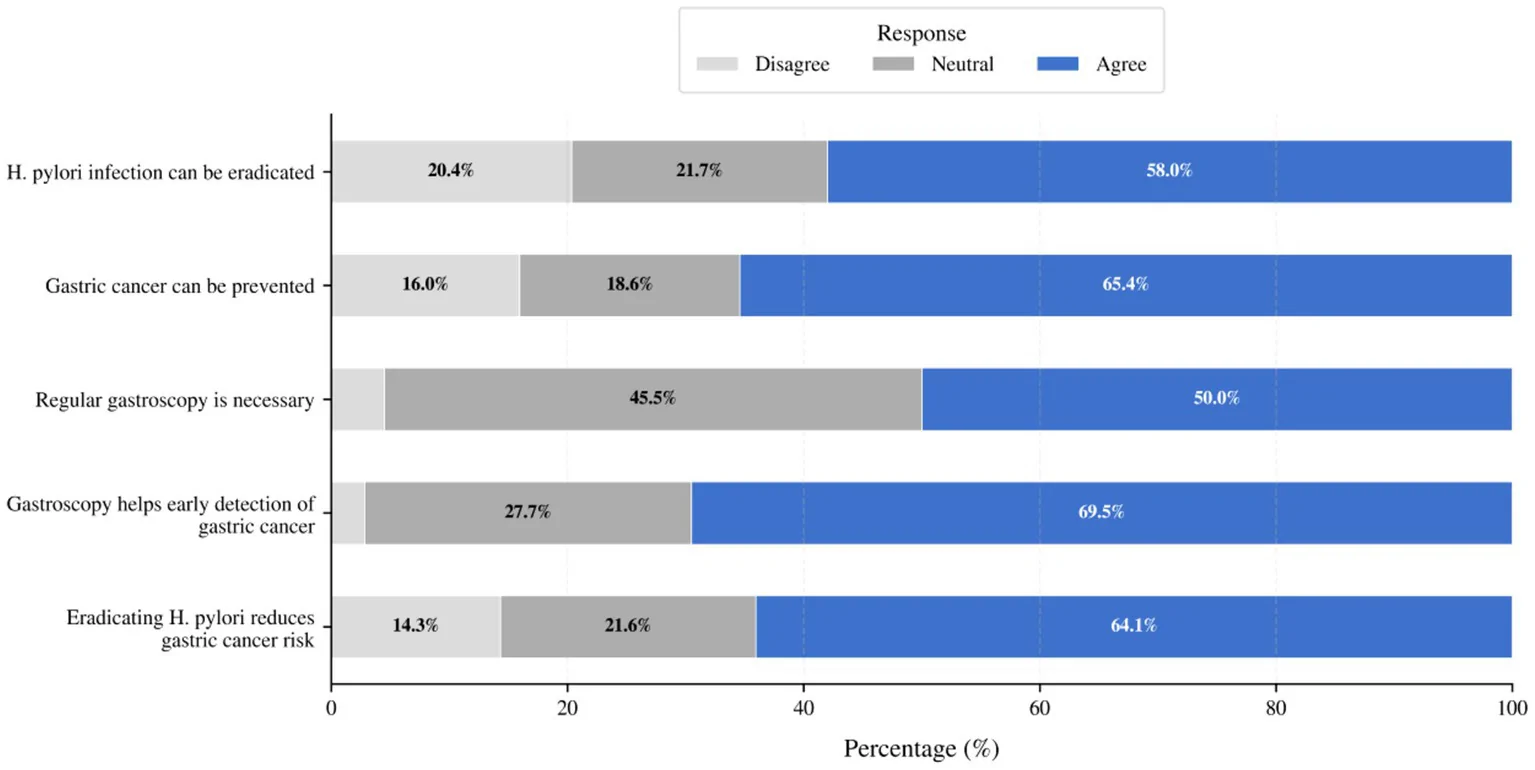
Participants’ beliefs regarding *Helicobacter pylori* infection and gastric cancer prevention (*N* = 1,911).

A total of 1,911 respondents demonstrated a favorable belief toward *H. pylori* infection and gastric cancer screening, with an overall standardized belief score of 0.749 on a 0–1 scale. Among the five items, the statement “UGE helps early detection of gastric cancer” achieved the highest standardized score (0.833), whereas “*H. pylori* infection can be eradicated” had the lowest score (0.688). This finding suggests a persistent gap in beliefs regarding the curability of *H. pylori* infection, indicating a potential target for future health education interventions. The distribution of responses for each belief item is presented in Figure 2.

#### 3.2.3 Previous UGE use

Regarding previous UGE use, 25.1% (480/1,911) of participants reported that they had previously undergone UGE. In contrast, 53.7% (1,027/1,911) had not undergone UGE but indicated willingness to consider it in the future, whereas 21.2% (404/1,911) reported that they did not plan to undergo UGE. Overall, actual uptake remained relatively low despite substantial stated willingness. Previous UGE use is presented in Table 1.

#### 3.2.4 Perceived barriers to UGE use

**Figure 3:**
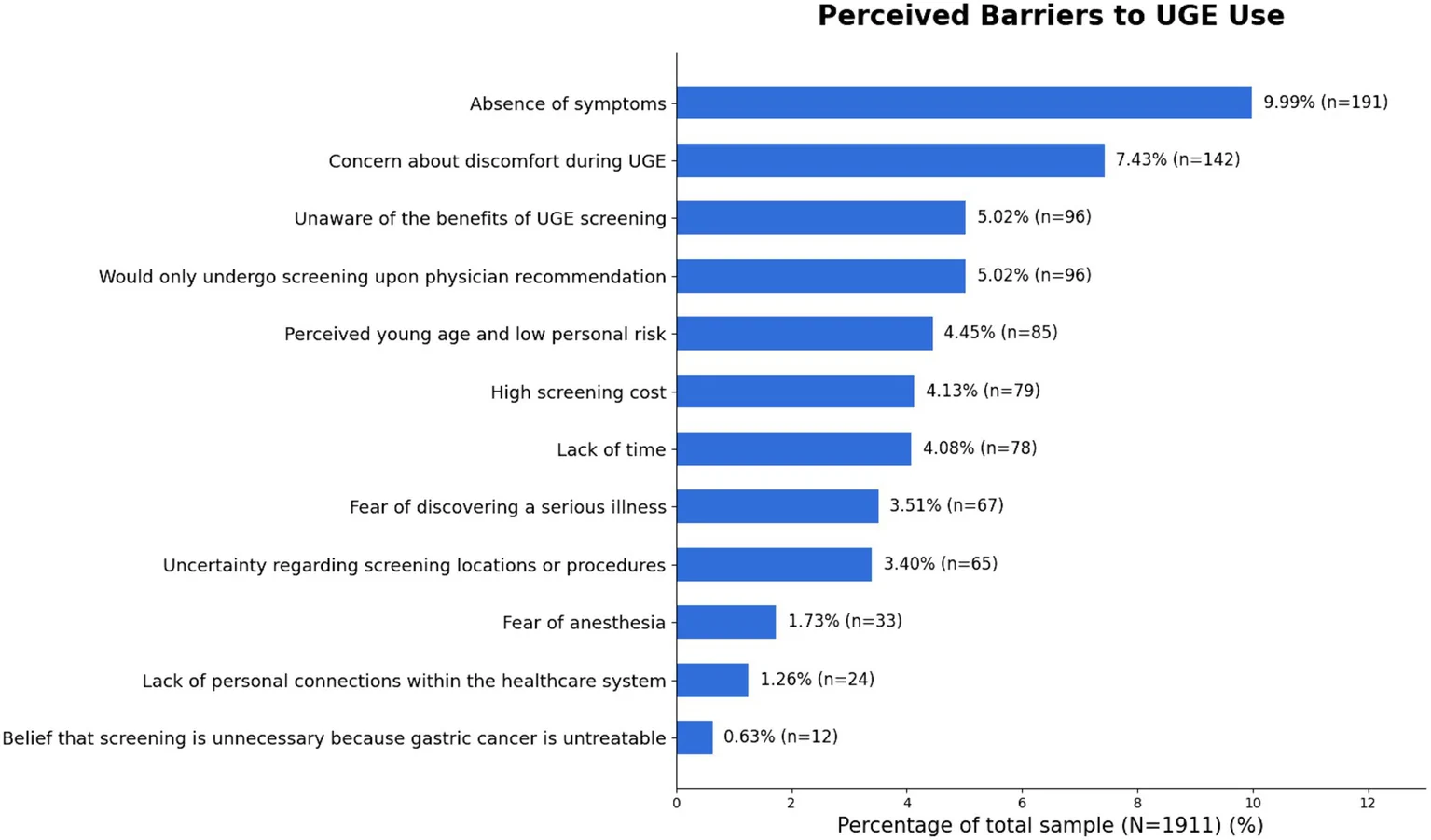
Frequency and percentage distribution of perceived barriers to UGE use among study participants (*N* = 1,911).

Among all participants, the most commonly reported perceived barrier to UGE use was the absence of symptoms (9.99%), followed by concern about discomfort during the procedure (7.43%). Limited awareness of screening benefits and reliance on physician recommendation were each reported by 5.02% of participants. Other barriers, including perceived low personal risk, screening costs, and lack of time, were reported by fewer than 5% of respondents. Detailed distributions of perceived barriers are presented in Figure 3.

### 3.3 Univariate analysis of factors associated with previous UGE use

Table 2 presents the univariate associations between participant characteristics and previous UGE use. Significant differences in previous UGE use were observed across marital status, education level, annual income, residence, self-reported *H. pylori* infection, gastrointestinal symptoms, first-degree family history of GC, having a friend or colleague with GC, and serving chopsticks/separate dining behaviors (all *p* < 0.05). No significant differences were observed by age, sex, family *H. pylori* infection, or knowledge score (all *p* > 0.05). Participants with previous UGE use had lower belief scores and perceived barrier scores than those without previous UGE use (both *p* < 0.001).

**Table 2:** Univariate analysis of factors associated with previous UGE use among community residents (*N* = 1,911).

Variable	Category	Previous UGE use		*χ*^2^/*t*/*F* (*p*)
		Yes	No	
Age	<45	349/1,427 (24.5%)	1,078/1,427 (75.5%)	1.173 (0.279)
	≥45	131/484 (27.1%)	353/484 (72.9%)	
Sex	Male	207/791 (26.2%)	584/791 (73.8%)	0.701 (0.402)
	Female	273/1,120 (24.4%)	847/1,120 (75.6%)	
Marital status	Unmarried	80/437 (18.3%)	357/437 (81.7%)	13.508 (<0.001)**
	Married	400/1,474 (27.1%)	1,074/1,474 (72.9%)	
Education level	Primary school or below	13/28 (46.4%)	15/28 (53.6%)	11.022 (0.026)*
	Junior high school or below	72/291 (24.7%)	219/291 (75.3%)	
	High school/Technical secondary	89/330 (27.0%)	241/330 (73.0%)	
	College/Bachelor	270/1,146 (23.6%)	876/1,146 (76.4%)	
	Master or above	36/116 (31.0%)	80/116 (69.0%)	
Annual income (CNY)	<30,000	159/784 (20.3%)	625/784 (79.7%)	20.792 (<0.001)**
	30,000–60,000	129/482 (26.8%)	353/482 (73.2%)	
	60,000–100,000	90/334 (26.9%)	244/334 (73.1%)	
	>100,000	102/311 (32.8%)	209/311 (67.2%)	
Residence	Urban	197/870 (22.6%)	673/870 (77.4%)	4.959 (0.026)*
	Rural	283/1,041 (27.2%)	758/1,041 (72.8%)	
*H. pylori* infection (self-reported)	Yes	138/293 (47.1%)	155/293 (52.9%)	87.524 (<0.001)**
	No	342/1,618 (21.1%)	1,276/1,618 (78.9%)	
Family *H. pylori* infection	Yes	131/480 (27.3%)	349/480 (72.7%)	1.611 (0.204)
	No/Unknown	349/1,431 (24.4%)	1,082/1,431 (75.6%)	
Gastrointestinal symptoms	Yes	282/848 (33.3%)	566/848 (66.7%)	52.890 (<0.001)**
	No	198/1,063 (18.6%)	865/1,063 (81.4%)	
First-degree relative with GC	Yes	98/148 (66.2%)	50/148 (33.8%)	141.707 (<0.001)**
	No	382/1,763 (21.7%)	1,381/1,763 (78.3%)	
Friend/colleague with GC	Yes	165/350 (47.1%)	185/350 (52.9%)	109.081 (<0.001)**
	No	315/1,561 (20.2%)	1,246/1,561 (79.8%)	
Use of serving chopsticks/separate dining at home	Yes	240/575 (41.7%)	335/575 (58.3%)	119.548 (<0.001)**
	No	240/1,336 (18.0%)	1,096/1,336 (82.0%)	
Use of serving chopsticks/separate dining when eating out	Yes	289/909 (31.8%)	620/909 (68.2%)	40.399 (<0.001)**
	No	191/1,002 (19.1%)	811/1,002 (80.9%)	
Knowledge score		0.61 ± 0.20 (*n* = 480)	0.60 ± 0.24 (*n* = 1,431)	−0.881 (0.378)
Belief score		0.70 ± 0.26 (*n* = 480)	0.77 ± 0.23 (*n* = 1,431)	5.226 (<0.001)**
Perceived barriers		0.0125 ± 0.11 (*n* = 480)	0.0519 ± 0.12 (*n* = 1,431)	198.713 (<0.001)**

Data are presented as *n*/N (%) or mean ± SD. *χ*^2^, Pearson chi-square statistic; *t*, independent-samples *t*-test statistic; *F*, one-way analysis-of-variance statistic; SD, standard deviation; GC, gastric cancer; CNY, Chinese Yuan; H. pylori, Helicobacter pylori; UGE, upper gastrointestinal endoscopy. *p < 0.05; **p < 0.001.

### 3.4 Multivariable logistic regression analysis

All prespecified variables listed in Section 2.3, except the perceived barrier score, were entered simultaneously into the multivariable logistic regression model. The perceived barrier score was evaluated separately as an intermediate construct in the Bayesian path analysis. The logistic regression results are presented in Supplementary Table S1.

In multivariable logistic regression analysis (*N* = 1,911), age <45 years versus ≥45 years was not associated with previous UGE use (aOR = 0.951, 95% CI 0.709–1.276, *p* = 0.739). Several other factors were independently associated with previous UGE use. Sociodemographic factors associated with lower odds of previous UGE use included being unmarried (aOR = 0.701, 95% CI 0.512–0.960, *p* = 0.027), lower household income (<30,000 CNY vs. >100,000 CNY: aOR = 0.548, 95% CI 0.383–0.783, *p* = 0.001), and urban residence (aOR = 0.726, 95% CI 0.566–0.931, *p* = 0.012).

Clinical and exposure factors, including self-reported *H. pylori* infection (aOR = 2.286, 95% CI 1.697–3.080, *p* < 0.001), family member with *H. pylori* infection (aOR = 1.343, 95% CI 1.001–1.802, *p* = 0.049), gastrointestinal symptoms (aOR = 1.666, 95% CI 1.320–2.103, *p* < 0.001), first-degree relative with GC (aOR = 4.648, 95% CI 3.135–6.891, *p* < 0.001), and friend/colleague with GC (aOR = 2.372, 95% CI 1.796–3.133, *p* < 0.001), were associated with higher odds of previous UGE use.

Behavioral factors such as using serving chopsticks or practicing separate dining at home (aOR = 0.407, 95% CI 0.306–0.542, *p* < 0.001) and when eating out (aOR = 0.684, 95% CI 0.521–0.899, *p* = 0.007) were associated with lower odds of previous UGE use after adjustment.

**Figure 4:**
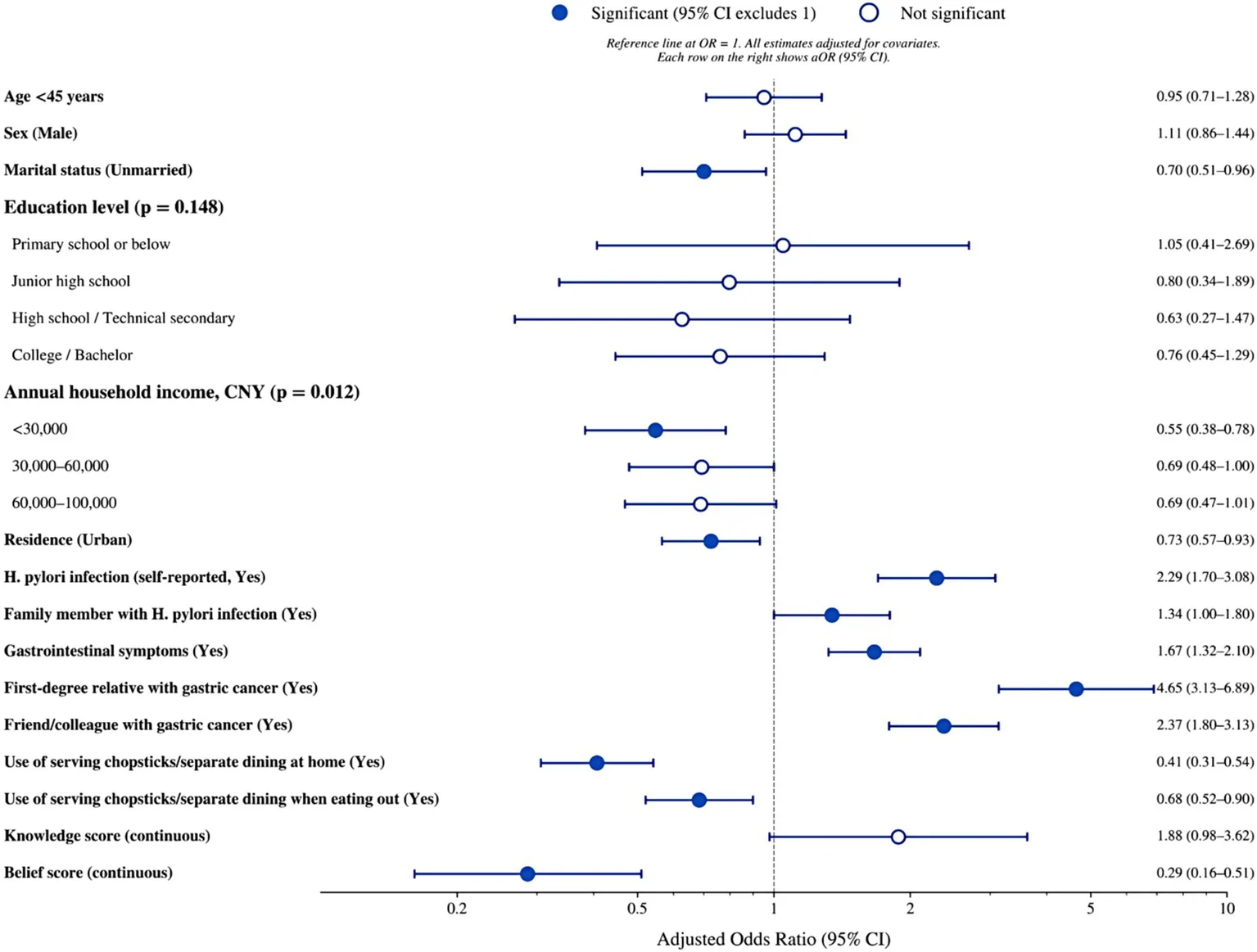
Forest plot of adjusted odds ratios for previous UGE use.

Among cognitive variables, higher knowledge score showed a borderline positive association with previous UGE use (aOR = 1.883, 95% CI 0.978–3.625, *p* = 0.058), whereas higher belief score was associated with lower odds of previous UGE use (aOR = 0.286, 95% CI 0.161–0.510, *p* < 0.001). The adjusted odds ratios and corresponding 95% CIs for all predictors are visually summarized in Figure 4. These associations should not be interpreted as causal effects.

### 3.5 Bayesian path analysis of indirect associations

Bayesian path analysis indicated that knowledge was positively associated with beliefs (B = 0.514, SE = 0.020, *p* < 0.001; *β* = 0.502, 95% CI [0.470, 0.532]), while beliefs were negatively associated with perceived barriers (B = −0.040, SE = 0.011, *p* < 0.001; *β* = −0.081, 95% CI [−0.125, −0.036]). Knowledge showed a modest positive direct association with previous UGE use (B = 0.572, SE = 0.163, *p* = 0.001; *β* = 0.126, 95% CI [0.056, 0.196]). Both beliefs and perceived barriers were associated with previous UGE use, with higher belief scores corresponding to lower previous UGE participation (Beliefs → Y: B = −0.987, SE = 0.154, *p* < 0.001; *β* = −0.223) and greater perceived barriers linked to lower previous UGE use (Barriers → Y: B = −1.962, SE = 0.308, *p* < 0.001; *β* = −0.220).

The indirect association of knowledge with previous UGE use through beliefs was significant (X → M1 → Y: effect = −0.507, SE = 0.082, p < 0.001, 95% CI [−0.669, −0.349]). A sequential indirect pathway through beliefs and perceived barriers was also observed (X → M1 → M2 → Y: effect = 0.040, SE = 0.013, *p* < 0.001, 95% CI [0.017, 0.069]). The total indirect association of knowledge was −0.467 (SE = 0.082, *p* < 0.001), whereas the direct association remained significant (B = 0.572, SE = 0.163, *p* = 0.001). The overall association of knowledge with previous UGE use was not statistically significant (B = 0.104, SE = 0.141, *p* = 0.227, 95% CI [−0.175, 0.384]). These indirect pathways should be interpreted as associative patterns rather than causal mediation.

**Figure 5:**
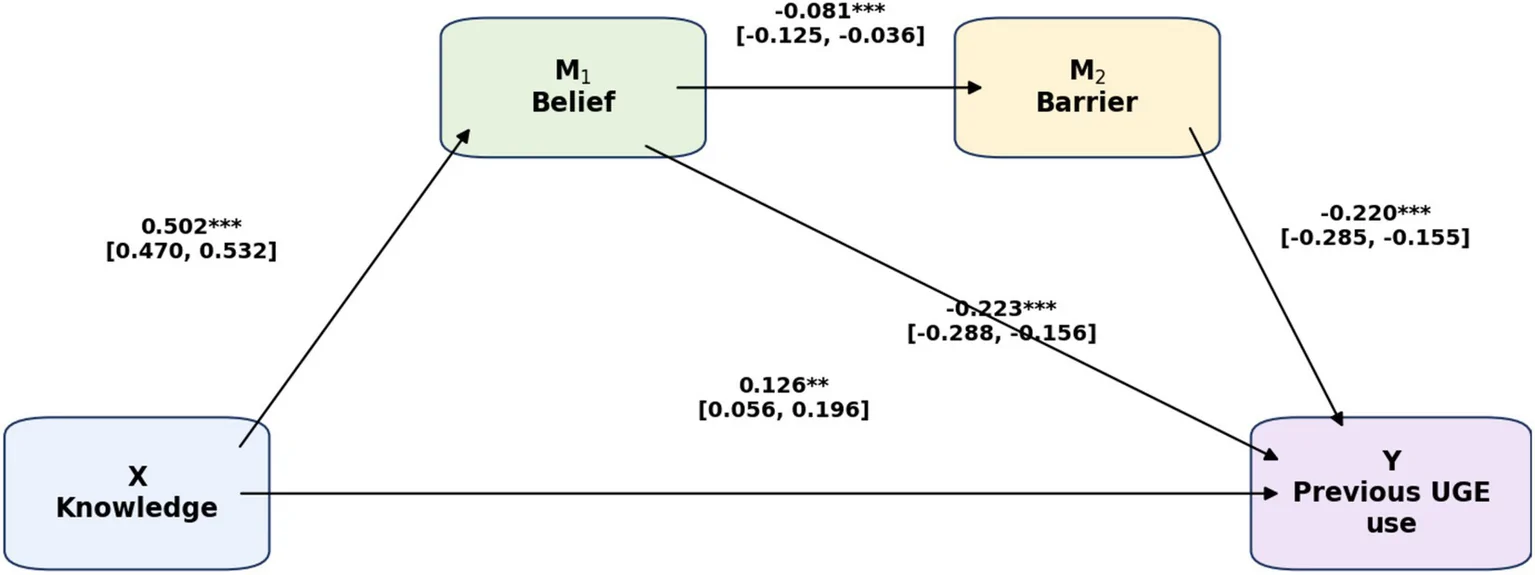
Bayesian path model showing direct and indirect association pathways among knowledge, beliefs, perceived barriers, and previous UGE use.

The model demonstrated acceptable fit to the data (Posterior Predictive *p*-value [PPP] = 0.307; 95% CI for chi-square difference: [−10.337, 16.900]). All path coefficients and estimated indirect associations are summarized in Table 3 and illustrated in Figure 5.

**Table 3:** Bayesian parameter estimates for direct and indirect association pathways in the path model (*N* = 1,911).

Pathway/parameter	Unstandardized (B)	Posterior S.D.	*p*-value	95% CI	Standardized (*β*)	95% CI (Std.)
Direct paths (structural model)						
X (Knowledge) → M₁ (Belief)	0.514	0.020	<0.001	[0.474, 0.554]	0.502	[0.470, 0.532]
M₁ (Belief) → M₂ (Barrier)	−0.040	0.011	<0.001	[−0.062, −0.018]	−0.081	[−0.125, −0.036]
X (Knowledge) → Y (Previous UGE use)	0.572	0.163	0.001	[0.251, 0.893]	0.126	[0.056, 0.196]
M1 (Belief) → Y (Previous UGE use)	−0.987	0.154	<0.001	[−1.290, −0.683]	−0.223	[−0.288, −0.156]
M2 (Barrier) → Y (Previous UGE use)	−1.962	0.308	<0.001	[−2.572, −1.365]	−0.220	[−0.285, −0.155]
Mediation effects (new parameters)						
Indirect Effect: X → M₁ → Y	−0.507	0.082	<0.001	[−0.669, −0.349]	–	–
Indirect Effect (Serial Chain): X → M₁ → M₂ → Y	0.040	0.013	<0.001	[0.017, 0.069]	–	–
Total Indirect Effect	−0.467	0.082	<0.001	[−0.630, −0.308]	–	–
Direct Effect	0.572	0.163	0.001	[0.251, 0.893]	–	–
Total Effect	0.104	0.141	0.227	[−0.175, 0.384]	–	–
Model fit indices	PPP = 0.307; 95% CI for *χ*^2^ difference: [−10.337, 16.900]					

Estimates were obtained using Bayesian path analysis with a probit link function. Standardized coefficients are STDYX standardized estimates. PPP = posterior predictive *p*-value; CI = credibility/credible interval. Because the study was cross-sectional, indirect pathways should be interpreted as indirect associations rather than causal mediation.

## 4 Discussion

### 4.1 Principal findings

Among 1,911 community residents in Henan Province, only one quarter reported previous UGE use. This proportion was low despite generally favorable beliefs about *H. pylori* infection and GC prevention and despite the fact that more than half of participants without previous UGE indicated willingness to consider it in the future. Knowledge was uneven rather than uniformly poor: participants were more likely to recognize broad prevention concepts, such as the role of UGE in early detection, than specific actionable information, including fecal-oral transmission routes of *H. pylori* and the age threshold for higher GC risk. The most frequently reported barriers were absence of symptoms and concern about endoscopic discomfort. In multivariable analysis, previous UGE use was more common among participants with self-reported *H. pylori* infection, gastrointestinal symptoms, first-degree family history of GC, or social exposure to GC, but was less common among participants with lower income. Bayesian path analysis further suggested that knowledge, beliefs, and perceived barriers were associated with previous UGE use in different directions. These findings indicate a gap between cognitive readiness and completed preventive action rather than a simple lack of awareness.

### 4.2 Relation to previous studies and the knowledge-action gap

The level of previous UGE use observed in this study is broadly consistent with Chinese community-based studies, but it remains lower than would be expected in a fully organized prevention pathway. Liu et al. reported that 15.2% of adults in Hunan had undergone gastric cancer screening, although most participants believed that screening was useful for early detection (30). Wang et al. found a screening rate of 21.5% among community residents in Shijiazhuang (17), and He et al. reported 23.4% screening uptake among Chinese adults aged 45 years or older (23). In southeastern China, a high-risk population study found moderate knowledge but relatively high intention to undergo screening, again suggesting that stated intention does not necessarily lead to attendance (15). These comparisons are consistent with our finding that favorable beliefs and willingness coexist with low completed UGE use.

The content of the knowledge deficit also deserves attention. In the present study, the weakest areas were not general recognition of GC prevention, but knowledge that can guide action, including routes of *H. pylori* transmission, eradication-related concepts, and eligibility for endoscopic evaluation. A Chinese survey on *H. pylori* screening similarly found poor public knowledge but generally positive attitudes toward testing; being asymptomatic and not knowing about testing were the main reasons for reluctance to undergo screening (31). These findings suggest that health education should move beyond broad statements that GC is preventable and should instead provide concrete guidance on who should be tested, how *H. pylori* infection is treated and confirmed, why asymptomatic risk still matters, and when endoscopic evaluation is appropriate.

### 4.3 Sociodemographic disparities and access to UGE

Age group (<45 vs. ≥45 years) was not associated with previous UGE use in either the univariate or multivariable analysis. This may appear unexpected because Chinese recommendations begin risk-based assessment and screening at 45 years (13, 14). However, the recommendations do not use age as a stand-alone indication; age is combined with factors such as *H. pylori* infection, family history, residence in a high-incidence area, adverse lifestyle, or relevant gastric disease. In addition, our outcome was previous UGE use rather than confirmed guideline-indicated screening and may include symptom-driven diagnostic examinations among younger adults. The predominance of participants aged <45 years (74.7%) and the broad binary categorization may also have limited the precision of age-related comparisons. The null association should therefore not be interpreted as evidence that age is unimportant for risk-based GC screening.

The association between socioeconomic position and previous UGE use is important for public health planning. In this study, lower household income was independently associated with lower odds of previous UGE use. Previous studies have also shown that screening knowledge and attendance are patterned by education, income, residence, health insurance, family history, previous gastric disease, and physician recommendation (16–18, 23, 24). More broadly, evidence from China indicates persistent social determinants in cancer screening, including unequal access to screening information, primary care referral, and diagnostic resources (25, 26). These disparities may be particularly consequential for UGE because the procedure requires facility access, appointment scheduling, preparation, time away from work, and tolerance of invasive testing.

The residence finding in our study should be interpreted cautiously. Urban residence was associated with lower odds of previous UGE use after adjustment, which is not the usual assumption if access is defined only by geographic proximity to hospitals. This result may reflect local sampling, different patterns of community mobilization, occupational time constraints in urban residents, or a higher likelihood that rural participants with symptoms or family history were referred through local health campaigns. It should not be taken to mean that rural access barriers are unimportant. Studies comparing urban and rural populations in China have reported differences in the distribution of high-risk groups and in compliance with endoscopic screening (25). Internationally, rural and socioeconomically disadvantaged populations may face longer travel distance, fewer endoscopy facilities, weaker continuity of care, and lower exposure to reliable prevention information. The IARC Latin America and Caribbean chapter highlights the continuing burden of GC and the need for organized prevention in underserved settings, including rural populations with poor survival after diagnosis (32).

International comparisons reinforce the importance of system organization. In Europe, implementation efforts such as GISTAR, EUROHELICAN, TOGAS, and HPSS are evaluating approaches that combine *H. pylori* screen-and-treat, pepsinogen testing, risk stratification, and integration with other screening infrastructures (33). However, IARC also noted that policy readiness and public awareness remain uneven across settings (33). In contrast, the Republic of Korea has a mature national GC screening program, and organized endoscopy-based screening has been associated with lower GC mortality compared with no screening (34). The implication is that individual knowledge and motivation are insufficient unless the health system provides a clear, affordable, and quality-assured route from risk recognition to testing, treatment, endoscopy, and follow-up.

### 4.4 Perceived barriers and cognitive-behavioral pathways

The leading barriers in this study, especially absence of symptoms and fear of endoscopic discomfort, suggest that many residents still regard gastroscopy primarily as a diagnostic test for symptomatic disease rather than as a preventive tool for high-risk asymptomatic individuals. This pattern is consistent with Chinese studies in which absence of symptoms, fear of discomfort, uncertainty about where to obtain screening, and insufficient physician recommendation were repeatedly reported as reasons for non-attendance (16, 17, 23, 30). A systematic review of gastric cancer screening barriers also identified lack of symptoms, fear of the procedure or result, cost, and embarrassment as common obstacles (21). These barriers are not merely informational deficits; they are practical and psychological constraints that can interrupt the pathway from knowledge to action.

The inverse association between the belief score and previous UGE use should therefore be interpreted as an associative and measurement-sensitive finding, not as evidence that favorable beliefs reduce screening. The belief scale was a brief KAP measure of general endorsement, whereas the Health Belief Model distinguishes perceived susceptibility, severity, benefits, barriers, cues to action, and self-efficacy (20). A participant may agree that UGE is useful while still perceiving low personal risk, fearing discomfort, lacking physician recommendation, or facing cost and time constraints. In addition, the outcome captured previous UGE use and could include symptom-driven diagnostic procedures, opportunistic examinations, surveillance, or preventive screening. The stronger associations with symptoms, *H. pylori* infection, and family history suggest that clinical salience may have triggered previous UGE more often than abstract preventive belief. The path analysis should therefore be read as evidence of an intention-action gap and possible construct-outcome mismatch rather than a causal mediation model.

### 4.5 Public health implications

Recent guidance on *H. pylori* screen-and-treat strategies provides a useful framework for translating these findings into practice. The 2025 IARC guidance and the 2026 IARC Working Group Report recommend population-based *H. pylori* screen-and-treat strategies for GC prevention in settings with sufficient need and readiness, while emphasizing needs assessment, sustainable governance, antibiotic stewardship, confirmation of eradication, quality indicators, information systems, and equity monitoring (6, 7). The Taipei Global Consensus II also supports *H. pylori* eradication as a major GC prevention strategy and highlights the rationale for family-based approaches because *H. pylori* infection often clusters within households (35). In our study, *H. pylori* infection among family members, a first-degree family history of GC, and having a friend or colleague with GC were associated with previous UGE use. These associative findings suggest that familial and social experiences may function as cues to action and may provide practical entry points for risk communication.

Family- and community-based strategies may help bridge the gap between individual awareness and sustained adherence. The HOPE Hp-GC Project in Latin America illustrates an implementation model that combines *H. pylori* testing and treatment with risk stratification and high-quality UGE for individuals at elevated risk (36). A related Latin American perspective argues that individual and family screen-to-treat approaches may be practical in high-burden settings when they are linked to endoscopy referral, eradication confirmation, and follow-up (37). In China, health-promotion programs could combine family risk communication, invitation or referral of household members for *H. pylori* testing, clinician recommendations for risk-appropriate UGE, and carefully designed peer or community narratives that normalize preventive evaluation without relying on fear appeals. A coherent pathway is needed from community risk assessment to *H. pylori* testing, standardized eradication treatment, confirmation of cure, navigation to UGE for eligible high-risk individuals, and surveillance for advanced precancerous conditions. Such an approach would address the fragmented implementation problem identified in this study, where knowledge, beliefs, perceived barriers, and completed preventive behavior were not aligned.

### 4.6 Limitations

Several limitations should be acknowledged. First, the cross-sectional design precludes conclusions about temporal order or causality. Bayesian path analysis was used to examine theoretically informed indirect associations, but these pathways should not be interpreted as causal mediation. Second, all information was self-reported, including *H. pylori* infection status and previous UGE use; recall bias and misclassification are possible. The self-reported *H. pylori* infection prevalence was also lower than many population-based estimates, probably because untested participants may have reported no infection or because the sample was relatively young. Third, previous UGE use could not be separated into preventive screening, symptom-driven diagnostic endoscopy, post-treatment follow-up, or surveillance, which limits interpretation of the behavioral outcome. Fourth, participants were recruited from one province using convenience and snowball sampling, and the online format may have selected individuals with better digital access and health interest. Fifth, 74.7% of participants were younger than 45 years, whereas current Chinese endoscopic screening recommendations primarily target eligible high-risk adults beginning at 45 years and generally focus on those aged 45–74 years (13, 14). This age distribution limits generalizability to the guideline-defined target population, and the binary age categorization may have obscured more detailed or nonlinear age-related patterns. Sixth, the questionnaire underwent internal validation, including expert review, cognitive debriefing, EFA, and reliability testing, but formal external validation in an independent population was not performed. Finally, the belief measure was brief and did not fully capture Health Belief Model constructs, physician recommendation, health literacy, endoscopy availability, insurance reimbursement, or previous clinical indications. Future longitudinal and implementation studies should oversample the guideline-targeted age group, examine age using more detailed categories or continuous nonlinear methods, distinguish screening from diagnostic endoscopy, and test whether combined education, barrier reduction, *H. pylori* screen-and-treat, family- and social-network risk communication, and risk-based endoscopy referral can improve adherence.

## 5 Conclusion

In conclusion, previous UGE use remained suboptimal among community residents despite generally favorable knowledge and beliefs regarding *H. pylori* infection and GC prevention. Knowledge, beliefs, and perceived barriers were associated with previous UGE use, but the cross-sectional design does not allow causal inference. The findings suggest that the challenge in GC prevention lies not only in increasing awareness but also in enabling preventive action through barrier reduction, physician recommendation, risk-based referral, and integration of population- and family-based *H. pylori* screen-and-treat strategies.

## Data Availability

The raw data supporting the conclusions of this article will be made available by the authors, without undue reservation.

## References

[1] Sung H Filho AM Laversanne M Ferlay J Siegel RL Soerjomataram I . Global cancer statistics 2024: GLOBOCAN estimates of incidence and mortality worldwide for 34 cancers in 186 countries. CA Cancer J Clin. (2024) 76:e70090. doi: 10.3322/caac.70090, 42417444 PMC13343830

[2] Han B Zheng R Zeng H Wang S Sun K Chen R . Cancer incidence and mortality in China, 2022. J Natl Cancer Cent. (2024) 4:47–53. doi: 10.1016/j.jncc.2024.01.006, 39036382 PMC11256708

[3] National Cancer Institute. Stomach Cancer Survival Rates and Prognosis. (2023). Available online at: https://www.cancer.gov/types/stomach/survival (Accessed July 12, 2026).

[4] Lee YC Chiang TH Chou CK Tu YK Liao WC Wu MS . Association between *Helicobacter pylori* eradication and gastric cancer incidence: a systematic review and meta-analysis. Gastroenterology. (2016) 150:1113–1124.e5. doi: 10.1053/j.gastro.2016.01.028, 26836587

[5] Pan KF Li WQ Zhang L Liu WD Ma JL Zhang Y . Gastric cancer prevention by community eradication of *Helicobacter pylori*: a cluster-randomized controlled trial. Nat Med. (2024) 30:3250–60. doi: 10.1038/s41591-024-03153-w, 39079993

[6] Park JY, editor. Population-Based *Helicobacter pylori* Screen-and-Treat Strategies for Gastric Cancer Prevention: Guidance on Implementation. IARC Working Group Reports, No. 12. Lyon: International Agency for Research on Cancer (2025). 40601795

[7] Park JY Lee YC Moayyedi P Lansdorp-Vogelaar I Camargo MC Tepes B . *Helicobacter pylori* screen-and-treat programs for gastric cancer prevention - IARC working group report. N Engl J Med. (2026) 394:1131–7. doi: 10.1056/NEJMsb2515372 41812202

[8] Zhang X Li M Chen S Hu J Guo Q Liu R . Endoscopic screening in Asian countries is associated with reduced gastric cancer mortality: a meta-analysis and systematic review. Gastroenterology. (2018) 155:347–354.e9. doi: 10.1053/j.gastro.2018.04.026, 29723507

[9] Xia C Li H Xu Y Guo G Yu X Wang W . Effect of an endoscopy screening on upper gastrointestinal cancer mortality: a community-based multicenter cluster randomized clinical trial. Gastroenterology. (2025) 168:725–40. doi: 10.1053/j.gastro.2024.11.025, 39706350

[10] Hibino M Hamashima C Iwata M Terasawa T. Radiographic and endoscopic screening to reduce gastric cancer mortality: a systematic review and meta-analysis. Lancet Reg Health West Pac. (2023) 35:100741. doi: 10.1016/j.lanwpc.2023.100741, 37424675 PMC10326711

[11] Dinis-Ribeiro M Libânio D Uchima H Spaander MCW Bornschein J Matysiak-Budnik T . Management of epithelial precancerous conditions and early neoplasia of the stomach (MAPS III): European Society of Gastrointestinal Endoscopy (ESGE), European Helicobacter and Microbiota Study Group (EHMSG) and European Society of Pathology (ESP) guideline update 2025. Endoscopy. (2025) 57:504–54. doi: 10.1055/a-2529-5025, 40112834

[12] Shah SC Wang AY Wallace MB Hwang JH. AGA clinical practice update on screening and surveillance in individuals at increased risk for gastric cancer in the United States: expert review. Gastroenterology. (2025) 168:405–416.e1. doi: 10.1053/j.gastro.2024.11.001, 39718517

[13] General Office of the National Health Commission of the People’s Republic of China. Gastric cancer screening and early diagnosis and treatment program (2024 edition). Beijing: National Health Commission (2024).

[14] He J Chen WQ Li ZS Li N Ren JS Tian JH . China guideline for the screening, early detection and early treatment of gastric cancer (2022, Beijing). Zhonghua Zhong Liu Za Zhi. (2022) 44:634–66. [in Chinese]. doi: 10.3760/cma.j.cn112152-20220617-00430, 35880331

[15] Huang Z Liu W Marzo RR Hu Z Wong LP Lin Y. High-risk population's knowledge of risk factors and warning symptoms and their intention toward gastric cancer screening in southeastern China. Front Public Health. (2022) 10:974923. doi: 10.3389/fpubh.2022.974923, 36033804 PMC9403326

[16] Huang Z Hu Z Wong LP Lin Y. Determinants of gastric cancer screening attendance in southeastern China: a cross-sectional study. BMJ Open. (2023) 13:e073925. doi: 10.1136/bmjopen-2023-073925, 37474189 PMC10360441

[17] Wang Q He XC Geng LX Jiang SL Yang CJ Xu KY . Public awareness of gastric cancer risk factors and screening behaviours in Shijiazhuang, China: a community-based survey. PLoS One. (2024) 19:e0311491. doi: 10.1371/journal.pone.0311491, 39374217 PMC11458051

[18] Aguiar-Ibanez R Mbous Y Sharma S Chakali R Chawla E. Barriers to cancer screening uptake and approaches to overcome them: a systematic literature review. Front Oncol. (2025) 15:1575820. doi: 10.3389/fonc.2025.1575820, 40842583 PMC12364675

[19] Kang K Bagaoisan MAP. Research status of the knowledge-attitude-practice theory model in gastric cancer prevention. Cureus. (2024) 16:e64960. doi: 10.7759/cureus.64960, 39161502 PMC11333023

[20] Alyafei A Easton-Carr R. "The health belief model of behavior change". In: StatPearls [Internet]. Treasure Island (FL): StatPearls Publishing (2026) 39163427

[21] Hatamian S Etesam S Mazidimoradi A Momenimovahed Z Salehiniya H. The barriers and facilitators of gastric cancer screening: a systematic review. J Gastrointest Cancer. (2021) 52:839–45. doi: 10.1007/s12029-021-00652-8, 34128198

[22] Wolf EJ Harrington KM Clark SL Miller MW. Sample size requirements for structural equation models: an evaluation of power, bias, and solution propriety. Educ Psychol Meas. (2013) 76:913–34. doi: 10.1177/0013164413495237, 25705052 PMC4334479

[23] He X Qi W Wang Q Zhao S. Knowledge and practice of early gastric cancer screening among adults aged >=45 years in China: a cross-sectional study. BMC Public Health. (2024) 24:3099. doi: 10.1186/s12889-024-20558-x, 39522036 PMC11549757

[24] Chang Y Cho B Son KY Shin DW Shin H Yang HK . Determinants of gastric cancer screening attendance in Korea: a multi-level analysis. BMC Cancer. (2015) 15:336. doi: 10.1186/s12885-015-1328-4, 25927821 PMC4424570

[25] Li H Cao MM Sun DQ He SY Yan XX Yang F . A comparative analysis of the distribution of the high-risk population of upper gastrointestinal cancer and endoscopic screening compliance in two urban areas and two rural areas in China. Zhonghua Zhong Liu Za Zhi. (2022) 44:531–9. doi: 10.3760/cma.j.cn112152-20210916-00707, 35754227

[26] Wang Z Xia Q Meng P Lu C Yang H Feng XL . Social determinants of health and cancer screening in China. Lancet Reg Health West Pac. (2024) 44:101043. doi: 10.1016/j.lanwpc.2024.101043, 38501078 PMC10945237

[27] Asparouhov T. Muthen B. Bayesian analysis using Mplus: technical implementation. Mplus Web Notes, No. 21. (2010). Available online at: https://www.statmodel.com/download/Bayes3.pdf

[28] World Health Organization. Advocacy, Communication and social Mobilization for TB Control: a guide to Developing Knowledge, Attitude and Practice Surveys. Geneva: World Health Organization (2008).

[29] Andrade C Menon V Ameen S Praharaj SK. Designing and conducting knowledge, attitude, and practice surveys in psychiatry: practical guidance. Indian J Psychol Med. (2020) 42:478–81. doi: 10.1177/0253717620946111, 33414597 PMC7750837

[30] Liu Q Zeng X Wang W Huang RL Huang YJ Liu S . Awareness of risk factors and warning symptoms and attitude towards gastric cancer screening among the general public in China: a cross-sectional study. BMJ Open. (2019) 9:e029638. doi: 10.1136/bmjopen-2019-029638, 31340970 PMC6661546

[31] Wang Y Zou J Hu L Wang YX Zou JY Hu LF . What is the general Chinese public's awareness of and attitudes towards *Helicobacter pylori* screening and associated health behaviours? A cross-sectional study. BMJ Open. (2022) 12:e057929. doi: 10.1136/bmjopen-2021-057929, 35078854 PMC8796245

[32] Riquelme A Camargo MC. "Gastric cancer prevention in Latin America and the Caribbean". In: Park JY, editor. Population-Based *Helicobacter pylori* Screen-and-Treat Strategies for Gastric Cancer Prevention: Guidance on Implementation. IARC Working Group Reports, No. 12. Lyon: International Agency for Research on Cancer (2025) 40601795

[33] Tepes B Leja M Matysiak-Budnik T Dinis-Ribeiro M Forman D Takens J . "Gastric cancer prevention efforts in Europe (EUROHELICAN, TOGAS, and HPSS projects)". In: Park JY, editor. Population-based *Helicobacter pylori* Screen-and-treat Strategies for gastric cancer Prevention: Guidance on Implementation. IARC Working Group Reports, No. 12. Lyon: International Agency for Research on Cancer (2025) 40601795

[34] Choi IJ. "Gastric cancer prevention in the Republic of Korea". In: Park JY, editor. Population-Based *Helicobacter pylori* Screen-and-Treat Strategies for Gastric Cancer Prevention: Guidance on Implementation. IARC Working Group Reports, No. 12. Lyon: International Agency for Research on Cancer (2025) 40601795

[35] Liou JM Malfertheiner P Hong TC Cheng HC Sugano K Shah S . Screening and eradication of *Helicobacter pylori* for gastric cancer prevention: Taipei global consensus II. Gut. (2025) 74:1767–91. doi: 10.1136/gutjnl-2025-336027, 40912906

[36] The Task Force HOPE Hp-GC Project Consortium. HOPE Hp-GC project - program implementation for primary and secondary prevention of gastric cancer in Latin America. Helicobacter. (2026) 31:e70109. doi: 10.1111/hel.70109, 42002545

[37] Portillo-Mino JD Uribe J Latorre G Pizarro M Pizarro M Palacios N . Individuals and family-screen-to-treat-based *Helicobacter pylori* eradication strategy for gastric cancer prevention: a Latin American perspective. Helicobacter. (2026) 31:e70110. doi: 10.1111/hel.70110, 42010149

