# Supplementary material for: Knowledge, beliefs, perceived barriers, and previous upper gastrointestinal endoscopy use regarding Helicobacter pylori and gastric cancer prevention among community residents: a cross-sectional study
Source: Front Public Health. 2026 Aug 14;14:1912334. doi: 10.3389/fpubh.2026.1912334 (PMC13522118; doi:10.3389/fpubh.2026.1912334)
Supplement: Supplementary file 1 [file Table_1.docx]

**Supplementary Table S1. Multivariable logistic regression analysis of factors associated with previous UGE use (N = 1,911).**

| **Variable** | **B** | **S.E.** | **Wald** | **p** | **aOR (95% CI)** |
| --- | --- | --- | --- | --- | --- |
| **Age <45 years (vs. ≥45 years)** | -0.050 | 0.150 | 0.111 | 0.739 | 0.951 (0.709–1.276) |
| **Sex (Male)** | 0.109 | 0.131 | 0.687 | 0.407 | 1.115 (0.862–1.442) |
| **Marital status (Unmarried)** | -0.355 | 0.160 | 4.918 | 0.027 | 0.701 (0.512–0.960) |
| **Education level** |  |  | 6.774 (4) | 0.148 |  |
| Primary school or below | 0.046 | 0.482 | 0.009 | 0.923 | 1.0479(0.407-2.694) |
| Junior high school or below | -0.227 | 0.441 | 0.265 | 0.607 | 0.797(0.336-1.892) |
| High school/Technical secondary | -0.466 | 0.435 | 1.15 | 0.284 | 0.627(0.268-1.471) |
| College/Bachelor | -0.273 | 0.271 | 1.013 | 0.314 | 0.761 (0.447–1.294) |
| **Annual household income (CNY)** |  |  | 11.033 (3) | 0.012 |  |
| <30,000 | -0.602 | 0.182 | 10.966 | 0.001 | 0.548 (0.383–0.783) |
| 30,000–60,000 | -0.367 | 0.188 | 3.816 | 0.051 | 0.693 (0.479–1.001) |
| 60,000–100,000 | -0.372 | 0.196 | 3.597 | 0.058 | 0.689 (0.469–1.012) |
| **Residence (Urban)** | -0.320 | 0.127 | 6.331 | 0.012 | 0.726 (0.566–0.931) |
| ***H. pylori* infection (self‑reported) (Yes)** | 0.827 | 0.152 | 29.678 | <0.001 | 2.286 (1.697–3.080) |
| **Family member with *H. pylori* infection (Yes)** | 0.295 | 0.150 | 3.864 | 0.049 | 1.343 (1.001–1.802) |
| **Gastrointestinal symptoms (Yes)** | 0.511 | 0.119 | 18.563 | <0.001 | 1.666 (1.320–2.103) |
| **First‑degree relative with gastric cancer (Yes)** | 1.536 | 0.201 | 58.639 | <0.001 | 4.648 (3.135–6.891) |
| **Friend/colleague with gastric cancer (Yes)** | 0.864 | 0.142 | 37.250 | <0.001 | 2.372 (1.796–3.133) |
| **Use of serving chopsticks/separate dining at home(Yes)** | -0.899 | 0.146 | 38.044 | <0.001 | 0.407(0.306-0.542) |
| **Use of serving chopsticks/separate dining when eating out(Yes)** | -0.38 | 0.139 | 7.406 | 0.007 | 0.684(0.521-0.899) |
| **Knowledge score (continuous)** | 0.633 | 0.334 | 3.584 | 0.058 | 1.883 (0.978–3.625) |
| **Belief score (continuous)** | -1.250 | 0.295 | 18.002 | <0.001 | 0.286 (0.161–0.510) |

Notes: aOR, adjusted odds ratio; CI, confidence interval; UGE, upper gastrointestinal endoscopy. All listed explanatory variables were entered simultaneously using the Enter method; no automated stepwise selection or univariate P-value screening was used. The perceived barrier score was excluded because it was modeled as an intermediate construct in the Bayesian path analysis. For all categorical variables, the last category served as the reference group: Married (marital status), Master or above (education), >100,000 CNY (annual income), rural residence, no self-reported H. pylori infection, no/unknown family H. pylori infection, no gastrointestinal symptoms, no first-degree relative with GC, no friend/colleague with GC, and no use of serving chopsticks/separate dining.
